# The genetic architecture of dog ownership: large-scale genome-wide association study in 97,552 European-ancestry individuals

**DOI:** 10.1093/g3journal/jkae116

**Published:** 2024-05-31

**Authors:** Tong Gong, Robert Karlsson, Shuyang Yao, Patrik K E Magnusson, Olesya Ajnakina, Andrew Steptoe, Laxmi Bhatta, Ben Brumpton, Ashish Kumar, Erik Mélen, 23andMe research team, Stella Aslibekyan, Adam Auton, Elizabeth Babalola, Robert K Bell, Jessica Bielenberg, Katarzyna Bryc, Emily Bullis, Daniella Coker, Gabriel Cuellar Partida, Devika Dhamija, Sayantan Das, Sarah L Elson, Nicholas Eriksson, Teresa Filshtein, Alison Fitch, Kipper Fletez-Brant, Pierre Fontanillas, Will Freyman, Julie M Granka, Karl Heilbron, Alejandro Hernandez, Barry Hicks, David A Hinds, Ethan M Jewett, Yunxuan Jiang, Katelyn Kukar, Alan Kwong, Keng-Han Lin, Bianca A Llamas, Maya Lowe, Jey C McCreight, Matthew H McIntyre, Steven J Micheletti, Meghan E Moreno, Priyanka Nandakumar, Dominique T Nguyen, Elizabeth S Noblin, Jared O'Connell, Aaron A Petrakovitz, G David Poznik, Alexandra Reynoso, Morgan Schumacher, Anjali J Shastri, Janie F Shelton, Jingchunzi Shi, Suyash Shringarpure, Qiaojuan Jane Su, Susana A Tat, Christophe Toukam Tchakouté, Vinh Tran, Joyce Y Tung, Xin Wang, Wei Wang, Catherine H Weldon, Peter Wilton, Corinna D Wong, Keng-Han Lin, Chao Tian, Tove Fall, Catarina Almqvist

**Affiliations:** Department of Medical Epidemiology and Biostatistics, Karolinska Institutet, Stockholm SE-171 77, Sweden; Department of Medical Epidemiology and Biostatistics, Karolinska Institutet, Stockholm SE-171 77, Sweden; Department of Medical Epidemiology and Biostatistics, Karolinska Institutet, Stockholm SE-171 77, Sweden; Department of Medical Epidemiology and Biostatistics, Karolinska Institutet, Stockholm SE-171 77, Sweden; Department of Behavioural Science and Health, Institute of Epidemiology and Health Care, University College London, London WC1E 7HBUK; Department of Biostatistics and Health Informatics, Institute of Psychiatry, Psychology and Neuroscience, King's College London, London SE5 8AF, UK; Department of Behavioural Science and Health, Institute of Epidemiology and Health Care, University College London, London WC1E 7HBUK; K.G. Jebsen Center for Genetic Epidemiology, Department of Public Health and Nursing, NTNU, Norwegian University of Science and Technology, Trondheim NO-7491, Norway; K.G. Jebsen Center for Genetic Epidemiology, Department of Public Health and Nursing, NTNU, Norwegian University of Science and Technology, Trondheim NO-7491, Norway; HUNT Research Centre, Department of Public Health and Nursing, NTNU, Norwegian University of Science and Technology, Levanger 7600, Norway; Clinic of Medicine, St. Olavs Hospital, Trondheim University Hospital, Trondheim NO-7030, Norway; Institute of Environmental Medicine, Karolinska Institutet, Stockholm SE-171 77, Sweden; Department of Clinical Sciences and Education, Södersjukhuset, Karolinska Institutet, Stockholm SE-118 83, Sweden; Institute of Environmental Medicine, Karolinska Institutet, Stockholm SE-171 77, Sweden; Department of Clinical Sciences and Education, Södersjukhuset, Karolinska Institutet, Stockholm SE-118 83, Sweden; Sachs' Children's Hospital, South General Hospital, Stockholm SE-118 61, Sweden; 23andMe, Inc., Sunnyvale, CA 94086, USA; 23andMe, Inc., Sunnyvale, CA 94086, USA; 23andMe, Inc., Sunnyvale, CA 94086, USA; Molecular Epidemiology, Department of Medical Sciences, and Science for Life Laboratory, Uppsala University, Uppsala SE-751 85, Sweden; Department of Medical Epidemiology and Biostatistics, Karolinska Institutet, Stockholm SE-171 77, Sweden; Pediatric Allergy and Pulmonology Unit at Astrid Lindgren Children's Hospital, Karolinska University Hospital, Stockholm SE-141 86, Sweden

**Keywords:** GWAS, dog ownership, replication, European ancestry, heritability

## Abstract

Dog ownership has been associated with several complex traits, and there is evidence of genetic influence. We performed a genome-wide association study of dog ownership through a meta-analysis of 31,566 Swedish twins in 5 discovery cohorts and an additional 65,986 European-ancestry individuals in 3 replication cohorts from Sweden, Norway, and the United Kingdom. Association tests with >7.4 million single-nucleotide polymorphisms were meta-analyzed using a fixed effect model after controlling for population structure and relatedness. We identified 2 suggestive loci using discovery cohorts, which did not reach genome-wide significance after meta-analysis with replication cohorts. Single-nucleotide polymorphism-based heritability of dog ownership using linkage disequilibrium score regression was estimated at 0.123 (CI 0.038–0.207) using the discovery cohorts and 0.018 (CI −0.002 to 0.039) when adding in replication cohorts. Negative genetic correlation with complex traits including type 2 diabetes, depression, neuroticism, and asthma was only found using discovery summary data. Furthermore, we did not identify any genes/gene-sets reaching even a suggestive level of significance. This genome-wide association study does not, by itself, provide clear evidence on common genetic variants that influence dog ownership among European-ancestry individuals.

## Introduction

Historically, humans domesticated wolves into dogs, and the cooperative relationship between dogs and humans has been documented where dogs have been mainly used to help humans herd other animals, hunt, and protect their homes (Lord *et al*. 2016). Today, dogs also provide companionship and social interactions with humans, not only as domestic pets but also in interventions for the rehabilitation of prisoners (Dell and Poole 2015) and in healthcare settings (Jones *et al*. 2019). Dog companionship is associated with important physical and psychosocial health benefits for humans across their life span. For example, dog ownership has been associated with decreased all-cause and cardiovascular mortality in a Swedish population-based epidemiological study (Mubanga *et al*. 2017), although other large studies did not find such a relationship (Ding *et al*. 2018). Dog owners, particularly those living alone and elderly, are found to be more physically active, less lonely, and have an improved perception of well-being (Müllersdorf *et al*. 2010; Gee *et al*. 2017). Children growing up with dogs have a lower risk of asthma (Fall *et al*. 2015, 2018) but not type 1 diabetes mellitus (Wernroth *et al*. 2017).

However, it is unclear whether the observed epidemiological associations between dog ownership and health outcomes are due to the dog adoption itself or the underlying differences in personality, health, genetics, and lifestyle between dog owners and non-dog owners. One possible explanation, which has not been addressed in previous literature, can be the potential pleiotropic effect or the genetic correlation influencing both the individual's choice of owning a dog and other traits. Evidence suggests that dog ownership has a strong genetic component. For example, we have previously provided some evidence of latent genetic components explaining 51–57% of population variation of dog ownership in a classical twin study (Fall *et al*. 2019). Further, twin and family studies have also reported equivalent heritability for traits such as personality (Vukasović and Bratko 2015), body mass index (Elks *et al*. 2012), and other human disease traits (Polderman *et al*. 2015). However, no previous studies have tried to identify genetic variation associated with dog ownership. Identifying the genetic factors that influence dog ownership will improve our understanding of (1) this complex human behavior trait; (2) shared genetic architecture; and (3) the potential causal relationship between dog ownership and other complex traits. Furthermore, a mechanistic understanding of dog ownership at the molecular level may also allow its beneficial effects on health outcomes to be attained through non-pharmacological intervention.

Therefore, our aim was to explore the molecular genetic architecture of dog ownership. Specifically, we aimed to identify loci associated with dog ownership in a genome-wide association study (GWAS) in a large sample, to estimate single-nucleotide polymorphisms (SNP)-based heritability and genetic correlation, and to provide gene-based functional analysis.

## Materials and methods

### Study participants and phenotype definitions

#### Discovery cohorts

We performed separate GWAS and a meta-analysis on dog ownership in 5 independent discovery cohorts of twins from the Swedish Twin Registry (STR) (Pedersen *et al*. 2002; Lichtenstein *et al*. 2006; Magnusson *et al*. 2013; Zagai *et al*. 2019). Two sub-cohorts from the Screening Across the Lifespan Twin (SALT) study, i.e. SALTY and TwinGene, included 6,053 and 10,911 participants born 1958 or earlier who previously participated in a telephone interview between 1998 and 2002 and donated saliva samples or blood samples. The Study of Twin Adults Genes and Environment (STAGE) cohort included 8,568 twins born 1959–1985 who participated in a web-based questionnaire and donated saliva samples. The Young Adult Twin Study in Sweden (YATSS) and the Child and Adolescent Twin Study in Sweden (CATSS) included 3,271 and 6,705 twins born 1986–1992 and 1992–1997 who participated in web-based questionnaires (YATSS) or parental telephone interviews (CATSS) and provided saliva samples.

Information on dog ownership in individuals older than 18 years was retrieved via individual data linkage to the 2 dog ownership registers held by the Swedish Board of Agriculture and the Swedish Kennel Club with available information from 2001 January 1 to 2016 December 31. As it is mandatory that every dog in Sweden is registered by their owners in the dog registers, the coverage of registered dogs is excellent (83%) (Statistics Sweden 2013). Dog ownership was defined by registration from either dog register during the period, enabling at least 1 year of follow-up for the youngest twins born in 1997. We also retrieved additional information on birth year, sex, and zygosity from the initial questionnaire sent to participants.

### Replication cohorts

Three cohorts were used as replication samples to assess SNP associations and the loci found in the meta-analysis of discovery cohorts: (1) the English Longitudinal Study of Aging (ELSA) including a cohort of individuals aged ≥50 years living in England in 2002 (Steptoe *et al*. 2013) and who also attended wave 5 follow-up survey (2010–2011); (2) wave 1 and wave 2 of genotyped individuals from the Swedish prospective birth cohort BAMSE (Swedish abbreviation for the Children, Allergy, Milieu, Stockholm, Epidemiology) who were born during 1994–1996 in Stockholm and followed at 1, 2, 4, 8, 12, 16, and 24 years of age (Wickman *et al*. 2002); and (3) the Trøndelag Health Study (HUNT), including people aged ≥20 years living in the Nord-Trøndelag region during 1984–1986, 1995–1997, 2006–2008, and 2017–2019 (Brumpton *et al*. 2022). A detailed description of each replication cohort regarding how recruitment was carried out, the definition of dog ownership, SNP genotyping, imputation, and statistical analysis, is provided in the Supplementary Methods and Supplementary Tables 1 and 2. Additionally, 23andMe, Inc. research participants (507,249 dog owners and 452,782 non-dog owners) were used to replicate the 2 suggested significant loci.

In short, the dog ownership information in ELSA was based on the responses to the wave 5 data collection (2010–2011) regarding the question “Do you keep any household pets inside your house/flat?” followed by items about specific pets (dog, cat, bird, other furry pet, or “other” type of pet). In BAMSE, dog ownership was defined as participants who responded to the questions “Are there/Have there been any pets at home? (Yes/No)” and “Which pet, including dogs, cats, or other (specify)” at 24 years of age. In HUNT, we retrieved information on dog ownership based on participants' responses to the question “Is there a dog in your home? (Yes/No).” In 23andMe, dog ownership information is defined as replying yes/no to “Do you own a dog?”.

### Genotyping, imputation, and association analyses

DNA from the saliva or blood of the study participants from the STR was extracted using the automatic systems of CheMagic (STAR Instrument, Hamilton Robotics) or the Puregene (Gentra Systems, Minneapolis, MN, USA) at the KI Biobank. SNP-based genome-wide genotyping was performed at the SNP&SEQ Technology Platform in Uppsala, Sweden, using the Illumina Infinium PsychArray-24 BeadChip (for CATSS, TwinGene, and SALTY) and the Illumina Global Screening Array Multi-Disease (GSA-MD) BeadChip (for YATSS and STAGE). Zygosity information encoded as a case/control phenotype was used for quality control. The genotyped samples were processed using the Ricopili pipeline (Lam *et al*. 2020) for quality control (QC). SNPs with missingness > 0.05 were removed. Samples failed QC due to any of the following: per-sample call rate < 0.98; excessive heterozygosity (FHET outside ±0.2); and sex mismatch. Markers failed SNP QC due to any of the following: per-SNP call rate < 0.98; invariant; Hardy–Weinberg disequilibrium (*P* < 1 × 10^−6^ in controls and *P* < 1 × 10^−10^ in cases); and difference in call rate between cases and controls >0.02. Post-QC genotypes were imputed to the Haplotype Reference Consortium panel release 1.1(HRC 1.1) (McCarthy *et al*. 2016). After QC and imputation, monozygotic co-twins' genotypes were then imputed from their paired genotyped sibling and added to the main dataset. Thus, the 5 STR sub-cohorts consisted of 31,566 individuals and ∼47 M available markers, in which over 7 M common variants with high imputation quality [INFO score > 0.6 and imputation dosage *R*^2^ > 0.8 and minor allele frequency (MAF) ≥ 1%, see Supplementary Table 1 for the steps of post-QC SNP filtering]. Similar QC and imputation processes on the genotype data from ELSA, BAMSE, and HUNT are described in detail in the Supplementary supplementary methods and Supplementary Tables 1 and 2. The genotyping, imputation, and GWAS of 23andMe samples were described elsewhere.

We carried out sub-cohort-specific GWAS of dog ownership for STR and HUNT participants after excluding individuals with non-European ancestry, using the 2-step SAIGE method developed by Zhou *et al*. (2018): step 1, fitting a null logistic mixed model for dog ownership, with adjustment for calendar year of birth (standardized for each cohort), sex, and population stratification using the top 3 principal components (PC) of genotypic variance and generating genetic relatedness matrix (GRM). The GRM was estimated from directly genotyped markers after applying linkage disequilibrium (LD) pruning to exclude markers with *r*^2^ > 0.1 in a sliding window covering 10,000 markers and a 1,000 marker increment. Step 2 is fitting a mixed model with respective SNP dosage data, genetic relationship matrix, and other covariates included in step 1. ELSA GWAS of dog ownership was analyzed using SNPTEST with adjustment for year of birth, sex, and population stratification (top 4 PCs) in the logistic model. BAMSE GWAS of dog ownership was analyzed using EPACTS with adjustment for sex, standardized calendar year of birth, and top 3 principal components for population stratification.

Meta-analyses of all 5 discovery cohorts and 3 replication cohorts were performed as inverse variance weighted fixed effect models using METAL (Willer *et al*. 2010). Multi-allelic variants were excluded from all cohorts before running the meta-analyses. Q–Q plots and genomic inflation factors were generated to assess the potential inflation of test statistics from residual population stratification for each cohort. The association estimates between SNPs and dog ownership were reported as the primary analysis of all individual cohorts and meta-analysis with Manhattan plots. Variants with *P*-values *P* < 5 × 10^−8^ were considered as genome-wide significant and *P* < 5 × 10^−7^ as suggestive significance in our meta-analyses. We also reported the heterogeneity of genetic effect estimates for selected variants (with *P* < 5 × 10^−6^) across cohorts using the *I*^2^ index (i.e. HetISq). The SNPs with the lowest association *P*-values from the meta-analyses were looked up in PhenoScanner to identify associated genes and functions and any previously reported associations with other diseases and traits using the GWAS Catalog (Staley *et al*. 2016; Buniello *et al*. 2019).

The 23andMe team performed the association analysis using logistic regression assuming an additive model for allelic effects adjusting for age, sex, top 5 PCs, and genotyping platforms and shared the results of 2 SNPs we requested for replication analysis based on a meta-analysis of the discovery cohorts.

### Heritability estimation using GWAS summary statistics

We estimated SNP-based heritability for dog ownership and genetic correlation with 30 complex human traits including lifestyle-related, physical measures, and common somatic and psychiatric conditions using LD score regression, i.e. regressing GWAS summary statistics on LD scores (Bulik-Sullivan *et al*. 2015). The summary statistics of complex trait GWAS that were used in the LD score regression were listed in the Supplementary Materials (see Supplementary Table 10). We report the false discovery rate (FDR)-corrected estimates based on the summary statistics from both GWAS meta-analyses (including and excluding replication cohorts) and the individual cohorts. Furthermore, the 23andMe team reported SNP-based heritability based on the LD score regression analyses as well.

### Functional annotation and gene-based analyses

Positional mapping, gene/gene-set-based analyses using generalized gene-set analysis of GWAS data (MAGMA v1.06), and tissue expression analysis (using Genotype-Tissue Expression v8 datasets) were performed using the functional mapping and annotation (FUMA) SNP2GENE pipeline/platform (Watanabe *et al*. 2017).

### Ethical statement

The study was approved by the Regional Ethical Review Authority in Stockholm, Sweden, and informed consents were received from all study participants in the STR and other replication cohorts. All data were pseudonymized prior to analyses.

## Results

### Characteristics of discovery and replication cohorts

The basic characteristics of the 5 discovery and 3 replication cohorts are reported in Table 1. The discovery cohorts consisted of 35,358 participants: 3,207 (9.1%) were identified as dog owners in the registers during 2001–2016, and 32,157 were non-dog owners. Compared with the latter, the dog owners were more frequently female, married, or cohabiting with a partner, with compulsory or high school education.

**Table 1.** Basic characteristics of discovery and replication cohorts.

	Discovery cohorts														
	CATSS (born 1992–1997)			YATSS (born 1986–1992)			STAGE (born 1959–1985)			TwinGene (born 1911–1959)			SALTY (born 1917–1958)		
	All	Non-dog owners	Dog owners	All	Non-dog owners	Dog owners	All	Non-dog owners	Dog owners	All	Non-dog owners	Dog owners	All	Non-dog owners	Dog owners
*N* (%)	6,705	6,491 (96.8)	214 (3.2)	3,271	2,986 (91.3)	285 (8.7)	8,568	7,401 (86.4)	1,167 (13.6)	107,61	9,934 (92.3)	827 (7.7)	6,053	5,339 (88.2)	714 (11.8)
Median (IQR) age	21 (20, 23)	21 (20, 23)	22 (21, 23)	28 (26, 30)	28 (26, 30)	28 (26, 30)	45 (38, 52)	45 (38, 52)	48 (41, 53)	74 (69, 81)	74 (69, 81)	71 (67, 76)	65 (61, 69)	65 (62, 69)	64 (60, 69)
Zygosity															
MZ	2,621 (39.1)	2,536 (39.1)	85 (39.7)	1,261 (38.5)	1,160 (38.9)	101 (35.4)	2,557 (29.8)	2,270 (30.7)	287 (24.6)	3,622 (24.8)	3,316 (24.7)	306 (25.9)	1,760 (29.1)	1,562 (29.3)	198 (27.7)
DZ same sex	2,232 (33.3)	2,167 (33.4)	65 (30.4)	928 (28.4)	839 (28.1)	89 (31.2)	2,790 (32.6)	2,411 (32.6)	379 (32.5)	5,605 (38.4)	5,168 (38.5)	437 (37.0)	2,139 (35.3)	1,902 (35.6)	237 (33.2)
Unknown	4 (0.1)	3 (0.0)	1 (0.5)	80 (2.5)	73 (2.4)	7 (2.5)	217 (2.5)	180 (2.4)	37 (3.2)	59 (0.4)	55 (0.4)	4 (0.3)	3 (0.1)	3 (0.1)	0
DZ opposite sex	1,848 (27.5)	1,785 (27.5)	63 (29.4)	1,002 (30.6)	914 (30.6)	88 (30.9)	3,004 (35.1)	2,540 (34.3)	464 (39.8)	5,304 (36.4)	4,871 (36.3)	433 (36.7)	2,151 (35.5)	1,872 (35.1)	279 (39.1)
Sex															
Male	3,393 (50.6)	3,346 (51.5)	47 (22.0)	1,251 (38.3)	1,207 (40.4)	44 (15.4)	3,509 (40.9)	3,202 (43.3)	307 (26.3)	6,753 (46.3)	6,241 (46.5)	512 (43.4)	2,906 (48.0)	2,618 (49.0)	288 (40.3)
Female	3,312 (49.4)	3,145 (48.6)	167 (78.0)	2,020 (61.7)	1,779 (59.6)	241 (84.6)	5,059 (59.1)	4,199 (56.7)	860 (73.7)	7,837 (53.7)	7,169 (53.5)	668 (56.6)	3,147 (52.0)	2,721 (51.0)	426 (59.7)
Civil status															
Missing	−	−	−	−	−	−	581 (6.78)	516 (6.97)	65 (5.57)	244 (1.67)	233 (1.74)	11 (0.93)	568 (9.38)	516 (9.66)	52 (7.28)
Married or cohabiting	−	−	−	−	−	−	5,653 (66.0)	4,761 (64.3)	892 (76.4)	11,300 (77.4)	10,353 (77.2)	947 (80.2)	4,407 (72.8)	3,874 (72.6)	533 (74.6)
Divorced or separated	−	−	−	−	−	−	274 (3.2)	223 (3.0)	51 (4.4)	1,112 (7.6)	1,025 (7.6)	87 (7.4)	476 (7.9)	416 (7.8)	60 (8.4)
Widowed	−	−	−	−	−	−	13 (0.15)	9 (0.12)	4 (0.34)	752 (5.15)	712 (5.31)	40 (3.39)	61 (1.01)	53 (0.99)	8 (1.12)
Living alone	−	−	−	−	−	−	2,047 (23.9)	1,892 (25.6)	155 (13.3)	1,182 (8.1)	1,087 (8.1)	95 (8.1)	541 (8.9)	480 (9.0)	61 (8.5)
Education															
Missing	−	−	−	71 (2.2)	63 (2.1)	8 (2.8)	735 (8.6)	623 (8.4)	112 (9.6)	88 (0.6)	85 (0.6)	3 (0.2)	477 (7.9)	435 (8.1)	42 (5.9)
Compulsory school	−	−	−	47 (1.4)	38 (1.3)	9 (3.2)	354 (4.1)	283 (3.8)	71 (6.1)	7,687 (52.7)	7,113 (53.0)	574 (48.6)	1,976 (32.6)	1,706 (32.0)	270 (37.8)
High school	−	−	−	1,420 (43.4)	1,261 (42.2)	159 (55.8)	3,436 (40.1)	2,872 (38.8)	564 (48.3)	2,794 (19.1)	2,538 (18.9)	256 (21.7)	1,598 (26.4)	1,418 (26.6)	180 (25.2)
University or higher	−	−	−	1,733 (53.0)	1,624 (54.4)	109 (38.2)	4,043 (47.2)	3,623 (49.0)	420 (36.0)	4,021 (27.6)	3,674 (27.4)	347 (29.4)	2,002 (33.1)	1,780 (33.3)	222 (31.1)
Average number of dog owned (SD)	−	−	1.1 (0.4)	−	−	1.3 (1.0)	−	−	1.5 (1.4)	−	−	1.7 (1.6)	−	−	1.8 (2.5)

	Replication cohorts								
	ELSA (born earlier–1952)			BAMSE (born 1994–1996)			HUNT (born 1897–1988)		
	All	Non-dog owners	Dog owners	All	Non-dog owners	Dog owners	All	Non-dog owners	Dog owners
*N* (%)	5,488	4,509 (82.2)	979 (17.8)	2,246	1,915 (85.3)	331 (14.7)	59,552	45,450 (76.3)	14,102 (23.7)
Median (IQR) age*	67 (63, 75)	68 (62, 76)	64 (60, 70)	24	24	24	53 (40, 67)	56 (40, 69)	49 (39, 58)
Zygosity									
MZ	−	−	−	−	−	−	−	−	−
DZ same sex	−	−	−	−	−	−	−	−	−
Unknown	−	−	−	−	−	−	−	−	−
DZ opposite sex	−	−	−	−	−	−	−	−	−
Sex									
Male	2479 (45.2)	2,030 (45.0)	449 (45.9)	1,045 (46.5)	924 (48.2)	121 (36.5)	27,362 (46.0)	20,771 (45.7)	6,591 (46.7)
Female	3,009 (54.8)	2,479 (55.0)	530 (54.1)	1,201 (53.5)	991 (51.8)	210 (63.5)	32,190 (54.0)	24,679 (54.3)	7,511 (53.3)
Civil status									
Missing	0 (0.00)	0 (0.00)	0 (0.00)	−	−	−	−	−	−
Married or cohabiting	3,847 (70.1)	3,106 (68.9)	741 (75.7)	−	−	−	−	−	−
Divorced or separated	618 (11.3)	503 (11.2)	115 (11.7)	−	−	−	−	−	−
Widowed	725 (13.21)	638 (14.15)	87 (8.89)	−	−	−	−	−	−
Living alone	298 (5.4)	262 (5.8)	36 (3.7)	−	−	−	−	−	−
Education									
Missing	910 (16.6)	754 (16.7)	156 (15.9)	−	−	−	−	−	−
Compulsory school	1,611 (29.3)	1339 (29.7)	272 (27.9)	−	−	−	−	−	−
High school	1,290 (23.5)	1,037 (23.0)	253 (25.8)	−	−	−	−	−	−
University or higher	1,677 (30.6)	1,379 (30.6)	298 (30.4)	−	−	−	−	−	−
Average number of dog owned (SD)	−	−	−	−	−	−	−	−	−

*Age by December 2016 for the five discovery cohorts and age at study participation for replication cohorts.

### Identifying novel loci in discovery and replication cohorts

**Fig. 1.**
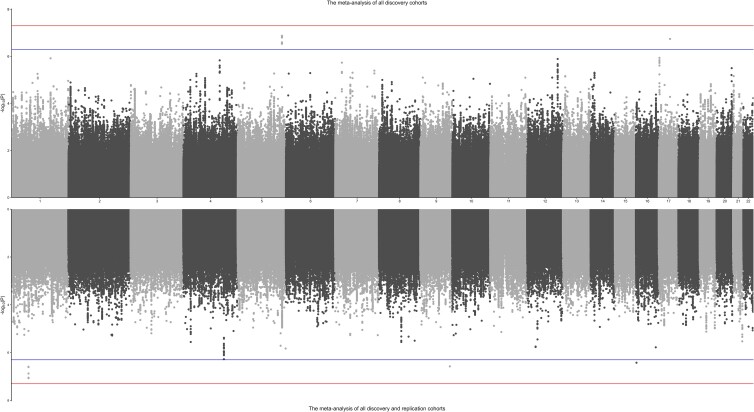
Manhattan plots displaying GWAS results for dog ownership from the meta-analysis of discovery cohorts with or without replication cohorts. The upper panel represents meta-analyzed GWAS results of discovery cohorts, and the lower panel represents results from discovery and replication cohorts. The red and blue lines indicate the *P*-value thresholds of 5 × 10^−8^ and 5 × 10^−7^, respectively.

After QC, exclusion of individuals with non-European ancestry, and GWAS analyses, association results for >7.4 million SNPs of 31,566 out of 35,358 twins (89%) were meta-analyzed using a fixed effect model. The ratio of the observed to the expected median association *χ*^2^ statistic (*λ*) was 1.02. Twelve SNPs located in chromosomes 5 and 17 (5q34 and 17q21.33) were suggestive with *P* < 5 × 10^−7^, but did not reach the genome-wide level of significance (see Supplementary Table 3 and Fig. 1). The association results for suggestive SNPs in the 5 individual twin cohorts showed similar effect sizes (see Supplementary Table 4 and Supplementary Fig. 1). Minimal population stratification was observed based on Q–Q plots and *λ*_GC_ (see Supplementary Fig. 2).

After including the 3 additional replication cohorts' eligible participants (*n* = 65,986), the meta-analyzed results based on 9,257,239 SNPs (of which 79.8% were presented in at least 7 out of 8 cohorts) are displayed in Supplementary Table 5. We did not observe any SNPs that reached genome-wide significance; however, we found 8 suggestive SNPs at 3 genetic risk loci (i.e. 1p31.1, 9q34.11, 16p13.3) with *P* < 5 × 10^−7^(Supplementary Tables 6 and 7). The nearest genes being looked up in the GWAS Catalog were *LRRC7*, *NCS1*, *AL360004*, and *HAGH* (Supplementary Table 6). The associations of the suggestive SNPs with dog ownership in the discovery cohorts did not appear as significant in the replication cohorts (Supplementary Table 4 and Supplementary Fig. 3). Neither did we observe the loci on chromosome 5 being associated with dog ownership in the 23andMe research participants (Supplementary Table 8).

### Heritability estimates

**Fig. 2.**
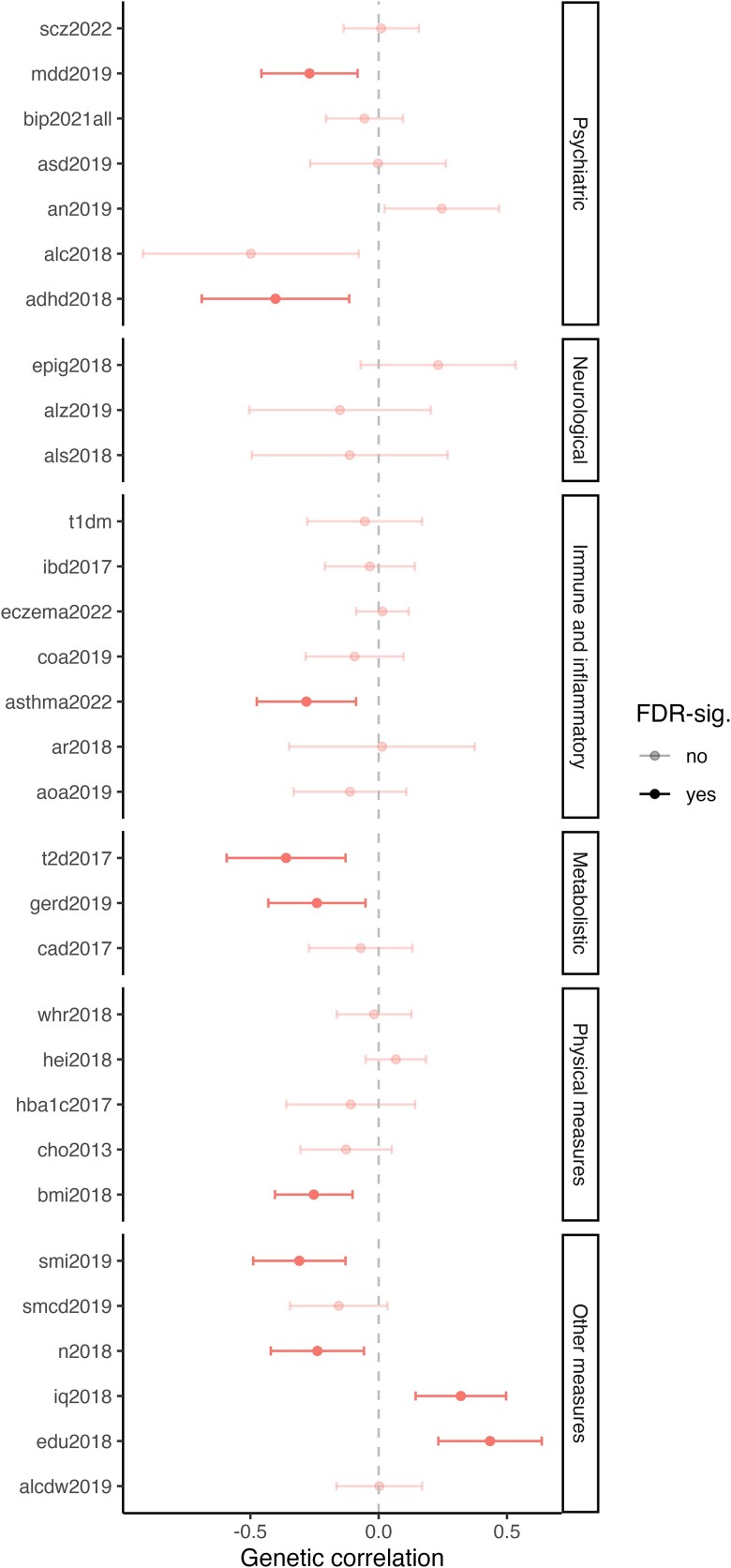
Genetic correlation between dog ownership (using summary data from the meta-analysis of discovery cohorts) and 30 complex traits.

We observed a SNP-based heritability estimate for dog ownership to be *h*^2^_SNP_ = 0.123 (CI 0.038–0.207) based on all discovery cohorts and the liability scale adjustment (Supplementary Table 9). Furthermore, we investigated the genetic correlation of dog ownership with 30 complex traits (see Supplementary Tables 10 and 11) by using LD score regression. After controlling for the FDR, there was a moderate correlation between dog ownership with educational attainment (*r_g_* = 0.43, *P* = 2.4 × 10^−5^), attention deficit hyperactivity disorder (ADHD) (*r_g_* = −0.40, *P* < 0.0062), correlations with type 2 diabetes(*r_g_* = −0.36, *P* < 0.0023), smoking initiation (*r_g_* = −0.31, *P* < 0.0008), neuroticism (*r_g_* = −0.24, *P* < 0.01), major depressive disorder (*r_g_* = −0.27, *P* < 0.005), intelligence (*r_g_* = 0.32, *P* < 0.0004), gastroesophageal reflux disease (*r_g_* = −0.24, *P* < 0.013), body mass index (*r_g_* = −0.25, *P* < 0.0044), and asthma (*r_g_* = −0.28, *P* < 0.0044, see Fig. 2 and Supplementary Table 11).

However, after adding the replication cohorts, the heritability estimates for dog ownership were reduced to *h*^2^_SNP_ = 0.008 (CI 0.004–0.018, Supplementary Table 9). Liability scale adjustment based on the population and sample prevalence did not seem to change the heritability estimates (*h*^2^_SNP_ = 0.018, CI −0.002 to 0.039), which was similar to the 23andMe heritability estimates (*h*^2^_SNP_ = 0.031, CI 0.029–0.033, Supplementary Table 9). Genetic correlations with tested 30 complex traits were no longer significant after FDR correction (Supplementary Fig. 4 and Supplementary Table 11).

### Functional annotation and gene-based analyses

Lastly, we examined whether aggregating suggestive SNP associations from the meta-analyzed results of all cohorts to the nearest genes (i.e. *LRRC7*, *NCS1*, *AL360004.1*, and *HAGH*) and linking them to tissue expressions might provide biological insights for dog ownership. We did not identify any target genes/gene-set elements that were significantly correlated with dog ownership after FDR correction (Supplementary Table 12 and 13 and Supplementary Fig. 5). Furthermore, the tissue expression analysis for sets of the mapped genes did not reveal any significantly enriched tissue types (Supplementary Fig. 6).

## Discussion

In this study, we performed the largest GWAS (*n* = 97,552) to date on dog ownership phenotype in European-ancestry individuals from 5 large discovery cohorts in Sweden, meta-analyzed with external European-ancestry cohorts from the United Kingdom and Norway. We found no common variants associated with dog ownership that reached the genome-wide significance threshold. Six suggestive loci were discovered in relation to dog ownership, and a weak SNP-based heritability estimate was observed. Moderate genetic correlations between dog ownership with complex traits including educational attainment and ADHD were identified, which indicates some evidence of shared genetic signatures, albeit wider confidence intervals when being replicated. Furthermore, the null result of the gene-based analyses has broadened the scope of our understanding of the genetic architecture of dog ownership to some extent.

Our study presents the first GWAS analysis focused on the trait of dog ownership. The primary finding is that no genetic locus reached genome-wide significance for dog ownership in the meta-analysis, including discovery and replication sets. This null finding can be due to phenotypic heterogeneity or misclassification, i.e. different dog ownership definitions in the discovery and replication cohorts. For example, the replication cohorts' measurement of dog ownership is based on the self-reported answers to “Are there dogs at home,” which does not necessarily indicate that the participant is the actual dog owner. The twin data are based on 2 dog ownership registers, which means that an individual within the household has to be motivated to register as the owner and be responsible for the dog. We could not access data on spouses/cohabiting partners to define the phenotype based on household dog ownership, which is likely to explain the lower observed prevalence of dog ownership in the STR cohorts compared to the general population. In this case, the observed results when leveraging large samples from different geographic areas (United Kingdom and Norway) could be diluted or biased toward null (Burstein *et al*. 2023). Furthermore, misclassification and changes in the dog ownership status over time might have skewed the phenotype distribution, biasing GWAS results toward the null. The dog ownership ascertainment among the younger and older twins was likely to be the main cause of these biases, as the parents of younger twins could have been the “registered” owners but not captured in CATSS or YATSS compared to self-reported dog ownership in BAMSE (prevalence 3–9% vs 15%). The longitudinal data from the dog ownership registers, with excellent population coverage from 2001 and onward, may not account for some of the previous owners in this study (8% in TwinGene vs 12% in SALTY).

Furthermore, as a primary parameter of the genetic architecture, the SNP-based heritability we identified was low (∼1.8%) in all discovery and replication samples and in 23andMe research participants (∼3.1%). This is comparable to the heritability estimates for some complex diseases such as Alzheimer's disease (∼3.1%) (Andrews *et al*. 2023). The presented “missing heritability” here was in line with findings from other complex diseases, highlighting the multifactorial etiology (Levinson *et al*. 2014). Even with the SNP-based heritability estimate of ∼12% for the discovery cohorts after adjusting for the liability scale, this is still much lower than the broad-sense heritability estimates of 51–57% for dog ownership in the same samples (Fall *et al*. 2019). There are several potential explanations for the “missing heritability.” First, gene–environment interactions where the effect of some genetic loci on the population variation of dog ownership could be dependent on certain environmental exposures (e.g. living area characteristics that could facilitate dog ownership) (Young 2019). Second, all common variants with small effects remain hidden in the noise based on the current sample size or the phenotype misclassification mentioned above, as we observed a dilution of both SNP-based heritability and genetic correlations when using the summary data based on the discovery and replication cohorts. Third, imputed data allowed us to evaluate the associated common variants that are not directly genotyped. However, rare variants may contribute to the heritability estimated the from classical twin model, but their effects could much less likely be captured in the current GWAS, which relied on the HRC reference panel. Fourth, the twin heritability can be overestimated due to the gene–gene and gene–environmental interaction, violation of equal environment assumptions (e.g. presence of assortative mating) (Border *et al*. 2022), and common genetic components determining dog ownership are minimal.

The possible genetic correlation underlying dog ownership and other complex traits including educational attainment, ADHD, type 2 diabetes, smoking initiation, neuroticism, major depressive disorder, intelligence, gastroesophageal reflux disease, body mass index, and asthma have been observed from epidemiological studies (Saunders *et al*. 2017; Zijlema *et al*. 2019). However, the phenotypic correlations are inconsistent, with reported opposite directions on some associations. Should these findings not be attributable to false positives, the genetic overlap may suggest potential pleiotropic effects of dog ownership with these somatic and mental health outcomes. Whether these findings could be translated into a causal relationship still deserves further investigations with powerful SNPs as instrumental variables.

The major strength of our study is the good coverage of dog ownership data in the Swedish general population and the capacity to link with the Swedish Twin Registers. However, there are several important limitations that are worth mentioning. First, we could not rule out misclassification with regard to the exposure time of dog ownership, which was defined as ever versus never, assuming this is a heritable trait. Furthermore, dog ownership information could be misclassified if the dog is registered under the partner's name, for which we lack information. Ideally, we would like to consider a time-to-event GWAS, accounting for censoring, changes in dog ownership over time/at different ages. Second, our findings, based on genotyped data from European populations, may not be generalizable to populations with other ancestries. Third, adjustments for population stratification based on common variants may be insufficient. Further investigations using family-based association analyses or PC adjustment of rare variants or identity by descent in even larger GWAS will be beneficial.

In conclusion, our meta-analysis of GWAS does not, by itself, provide clear evidence on common variants that influence dog ownership among European-ancestry individuals. The “missing heritability” should be further investigated in larger samples.

## Supplementary Material

jkae116_Supplementary_Data

## Data Availability

GWAS summary statistics with discovery and replication cohorts are uploaded on the journal figshare: https://doi.org/10.25387/g3.25713189. 23andMe participants provided informed consent and volunteered to participate in the research online, under a protocol approved by the external AAHRPP-accredited IRB, Ethical & Independent (E&I) Review Services. As of 2022, E&I Review Services is part of the Salus IRB (https://www.versiticlinicaltrials.org/salusirb). The full GWAS summary statistics for the 23andMe discovery dataset will be made available through 23andMe to qualified researchers under an agreement with 23andMe that protects the privacy of the 23andMe participants. Datasets will be made available at no cost for academic use. Please visit https://research.23andme.com/collaborate/#dataset-access for more information and to apply to access the data. Supplemental material available at G3 online.
