# Supplementary material for: The genetic architecture of dog ownership: large-scale genome-wide association study in 97,552 European-ancestry individuals
Source: G3 (Bethesda). 2024 May 31;14(8):jkae116. doi: 10.1093/g3journal/jkae116 (PMC11304603; doi:10.1093/g3journal/jkae116)
Supplement: jkae116_Supplementary_Data [file jkae116_supplementary_data.zip › Supplemental_Methods_and_Figures_G3-2024-405045.docx]

**Supplementary materials to the manuscript**

The genetic architecture of dog ownership: large-scale genome-wide association study in 97,552 European-ancestry individuals

Tong Gong^1^, Robert Karlsson^1^, Shuyang Yao^1^, Patrik Magnusson^1^, Olesya Ajnakina^2,3^, Andrew Steptoe^2^, Laxmi Bhatta^4^, Ben Brumpton^4,5,6^, Ashish Kumar^7^, Erik Mélen^7,8,9^, 23andMe research team^10^, Tove Fall^11^, Catarina Almqvist^1,12^

1. Department of Medical Epidemiology and Biostatistics, Karolinska Institutet, Stockholm, Sweden

2. Department of Behavioural Science and Health, Institute of Epidemiology and Health Care, University College London, London, United Kingdom

3. Department of Biostatistics & Health Informatics, Institute of Psychiatry, Psychology and Neuroscience, King’s College London, London, United Kingdom

4. K.G. Jebsen Center for Genetic Epidemiology, Department of Public Health and Nursing, NTNU, Norwegian University of Science and Technology, Trondheim, Norway

5. HUNT Research Centre, Department of Public Health and Nursing, NTNU, Norwegian University of Science and Technology, Levanger 7600, Norway

6. Clinic of Medicine, St. Olavs Hospital, Trondheim University Hospital, Trondheim, Norway

7. Institute of Environmental Medicine, Karolinska Institutet, Stockholm, Sweden

8. Department of Clinical Sciences and Education, Södersjukhuset, Karolinska Institutet, Stockholm, Sweden

9. Sachs’ Children’s Hospital, South General Hospital, Stockholm, Sweden

10. 23andMe, Inc., Sunnyvale, CA, USA

11. Molecular Epidemiology, Department of Medical Sciences, and Science for Life Laboratory, Uppsala University, Uppsala, Sweden

12. Pediatric Allergy and Pulmonology Unit at Astrid Lindgren Children’s Hospital, Karolinska University Hospital, Stockholm, Sweden

**Corresponding author**

Tong Gong, PhD

Department of Medical Epidemiology and Biostatistics,

Karolinska Institutet,

Box 281,

171 77 Stockholm

Sweden

Tel: +46 8 524 85000

Fax: +46 8 31 49 75

Email: tong.gong@ki.se

Contents

[**Supplementary Methods** 3](#_Toc165290311)

[Individual cohort details used for replication analysis 3](#_Toc165290312)

[1. ELSA 3](#_Toc165290313)

[2. BAMSE 4](#_Toc165290314)

[3. HUNT 5](#_Toc165290315)

[**Supplementary figures** 7](#_Toc165290316)

[Figure S1. Manhattan plots by each discovery cohort 7](#_Toc165290317)

[Figure S2. QQ-plots of each discovery cohort and meta-analysed all discovery cohorts 8](#_Toc165290318)

[Figure S3. Manhattan plots displaying GWAS results for dog ownership from the meta-analysis of discovery and replication cohorts separately. Upper panel represents meta-analyzed GWAS results of discovery cohorts and lower panel represents results from replication cohorts. Red and blue lines indicate the p-value thresholds at 5e-8 and 5e-7 respectively. 9](#_Toc165290319)

[Figure S4. Genetic correlation coefficients and 95% confidence intervals between dog ownership (using data from meta-analyzed discovery and replication cohorts) and 30 complex traits. 10](#_Toc165290320)

[Figure S5. Mahattan Plot of gene-based test 11](#_Toc165290321)

[Figure S6. Visualization of MAGMA tissue expression analysis. 12](#_Toc165290322)

[**Readme for the summary statistics** 13](#_Toc165290323)

[**Reference** 14](#_Toc165290324)

# **Supplementary Methods**

## Individual cohort details used for replication analysis

### 1. ELSA

#### Sample

We utilised data from the English Longitudinal Study of Ageing (ELSA), which is an ongoing large, multidisciplinary study of a nationally representative sample of the English population aged ≥50 years. The ELSA study started in 2002 (wave 1), with participants recruited from an annual cross-sectional survey that is designed to monitor the health of the general population. Ethical approval for each of the ELSA waves was granted by the National Research Ethics Service (London Multicentre Research Ethics Committee). All participants gave informed consent.

#### Phenotype measurement

For the purposes of the analyses, the study baseline is wave 5 (2010-11), when inquiries about pet ownership were first introduced into the study. Cohort members were asked “Do you keep any household pets inside your house/flat?” followed by items about specific pets (dog, cat, bird, other furry pet, or “other” type of pet). Information on pet ownership was available on 5488 ELSA participants for whom the quality-controlled genome-wide genotyping and dementia status during the follow-up were available; of these, 979 (17.8%) reported to own a dog. Of those who have reported to own a dog, 449 (45.9%) were men and 530 (54.1%) were women.

#### Genotyping, imputation, and statistical analysis

*Quality control*. The genome-wide genotyping was performed at University College London Genomics in 2013-2014. This involved genotyping ELSA participants of European ancestry using the Illumina HumanOmni2.5 BeadChips (HumanOmni2.5-4v1, HumanOmni2.5-8v1.3). Quality control was performed using PLINK(1), R and VCFtools(2). Samples were excluded if they had call rates <98%, deviated from the expected inbreeding coefficient (|F_het_|<0.2), had a sex discrepancy between reported and genotypic sex (based on inbreeding coefficients calculated from SNPs on the X chromosome), or were shown to be duplicates or related. Duplicated samples and cryptic relatedness between each pair of participants was evaluated using pairwise genome-wide estimates of three coefficients corresponding to the probabilities of sharing 0, 1 or 2 alleles between two individuals that are identical by descent (3) using estimating the identical by descent (IBD) probabilities(4). One of each pair of individuals with an IBD value of >0.2, which is halfway between the expected IBD for third- and second-degree relatives (5), was excluded at random. Samples were further removed based on suspected non-European ancestry. SNPs were excluded if the minor allele frequency (MAF) was <0.01%, if more than 2% of genotype data were missing and if the Hardy-Weinberg Equilibrium (HWE) *P*-value<10^−4^. Non-autosomal markers were also removed. In total, 7183 samples (96.9% of 7412 original cohort) and 1 372 240 (61.5% of 2230767) variants remained after these quality control steps.

*Genetic imputation.* To estimate genotypes that were not assayed, imputation was performed on the Michigan Imputation Server(6) running SHAPEIT for pre-phasing(7), and Minimac3 for imputation(8, 9) to the Haplotype Reference Consortium (HRC.r1-1.GRCh37)(6, 10); all variants aligned to human genome build 19 (hg19). After imputation, we required very high imputation quality (INFO>0.95), low missingness (<1%) for further quality control. We limited our analyses to variants genotyped or imputed with HWE *P*-value>10^−5^. We further applied stringent pruning to remove markers in high linkage disequilibrium (r^2^>0.1). After the sample quality control, 7 179 780 variants were retained for further analyses.

*Principal components analysis*. In order to investigate population structure, we chose less correlated SNPs for principal components analysis. The SNP pruning was performed following the procedure: i) consider a window of 50 SNPs, ii) calculate LD between each pair of SNPs in the window, iii) remove one of a pair of SNPs if the LD is greater than 0.5, iv) shift the window 5 SNPs forward and v) repeat the procedure. Altogether, 1083252 autosomal SNPs remained after the SNP pruning and were used to run principal components analysis. As a result, the top 10 principal components retained to account for any ancestry differences in genetic structures that could potentially bias the results(11).

*Genome-wide association analysis*. Genotype-phenotype association analyses were performed using SNPTEST(12) on imputed SNP dosage scores assuming an additive allelic effect. Adjustments for year of birth (standardised), sex, and population stratification (4 PCs) were included in the model.

### 2. BAMSE

*Sample prep and genotpying*

Within the BAMSE project, genotyping was done in two waves. Wave1 was done on the Illumina Human 610-quad array (Illumina, Inc., San Diego, CA). A total of 505 samples were genotyped (a subset of the study consisting of asthma cases and controls, out of which 488 samples were of good genotype quality. Wave2 was done on the Illumina Infinium Global Screening Array-24 v1.0 (GSA) BeadChip, where a total of 2387 samples were genotyped, out of which 2367 samples were of good genotype quality. Samples were further excluded if the 10 genetic principal components indicated a non-EU ethnic outlier after projection of the study samples on the 1000 Genomes reference sample.

*Imputation*

For imputation, samples were also excluded if their genotyping success rate was lower than 98% and SNPs were excluded for - Call rate < 98%; HWE P<1e-6 in controls and HWE P<1e-10 in cases; MAF < 0.01, determined using Ricopili-pipeline at MGH (version 8.0.0). This Ricopili pipeline used Sanger genotype imputation servers - using EAGLE2 for pre-phasing and phasing, and PBWT (Positional Burrows-Wheeler Transform) for genotype imputation, using the HRC reference panel version HRC1.1

*Principal components analysis*.

Additional 2 samples were removed for relatedness and 22 samples for population stratification (excluded samples were greater than 6 standard deviation from mean PCs of 1000G European reference samples) in wave1 while 54 samples were removed for relatedness and 115 samples for population stratification (excluded samples were greater than 6 standard deviation from mean PCs of 1000G European reference samples) in wave2. Thus, a remainder of 464 participants remained within wave1 and 2194 participants remained within wave2.

*Genome-wide association analysis*.

GWAS analysis was performed using EPACTS version v3.3.0. using logistic Wald test, on samples with available qualifying phenotype. Dog ownership was defined as 2a) parents who responded to the questions, “Are there /Have there been any pets at home?” and “Which pet, including dogs, cats, or other (specify)” from the any of the BAMSE initial or follow-up questionnaires (at the participants age of 0, 4, 8, 12 years old); and 2b) participants themselves who responded to the same question at 24 years of age. As per phenotype definitions, 365 samples qualified for analysis in wave1 and 1881 samples qualified for analysis in wave2. Additional covariates of sex, standardized calendar year of birth and top three principal components were used.

### 3. HUNT

The Trøndelag Health Study (HUNT) is a population-based study of ~125,000 participants, which invited the entire adult (≥20 years) population of northern Trøndelag(13, 14, 15). So far, four rounds of HUNT study has been conducted including HUNT1 (1984 to 1986, N=75,027, 86.8% of invited), HUNT2 (1995 to 1997, N=65,402, 69.7% of invited), HUNT3 (2006 to 2008, N=50,663, 54.0% of invited), and HUNT4 (2017 to 2019, N=56,042, 54.0% of invited)(13, 14, 15). All the adults were invited for questionnaires, interviews, clinical examinations, laboratory measurements, and/or storage of biological samples in at least one of four study rounds.

Information on dog ownership was retrieved from HUNT2 and HUNT3 based on the questionnaire “Are there any pets in your home?” where “dog” was one of the options.

The current analysis includes genetic data from ~90% (N=71,860) of participants from HUNT2 and HUNT3 who were genotyped by genome-wide SNP arrays in 2015 (16, 17). The genotyping and quality control metrics have been described elsewhere (17). In brief, one of three different Illumina HumanCoreExome arrays (HumanCoreExome12 v1.0, HumanCoreExome12 v1.1 and UM HUNT Biobank v1.0) were used for genotyping the HUNT2 and HUNT3 samples(17). Participants of European origin were previously defined by projecting principal components (PC) of HUNT participants into the Human Genome Diversity Project (HGDP) reference panel (18, 19). Only individuals of European ancestry were included in the study.

**Statistical analysis**

In the HUNT study, there is a large amount of relatedness between participants(17) therefore, to avoid the need to exclude related participants we used a method that accounts for the genetic relatedness and unbalanced case-control. We performed generalized mixed model association test (20). The models were adjusted for birth year, sex, study participation round (outcome was measured in HUNT2 and HUNT3), batch, and 4 principal components (PCs). Analyses were performed using R 4.0.3 (<http://www.r-project.org>) and SAIGE 0.43.1 software (20).

# **Supplementary figures**

## Figure S1. Manhattan plots by each discovery cohort


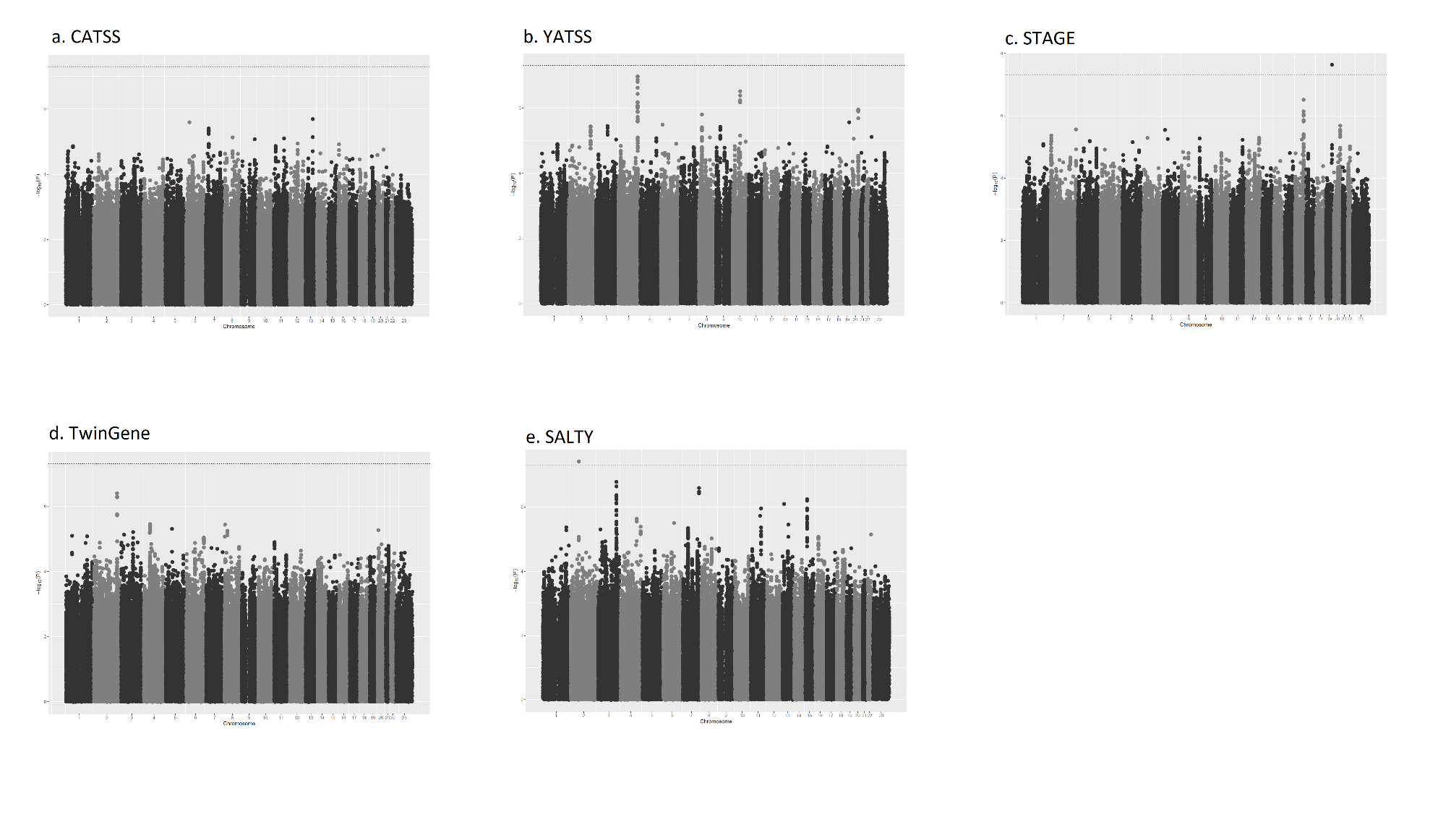


## Figure S2. QQ-plots of each discovery cohort and meta-analysed all discovery cohorts


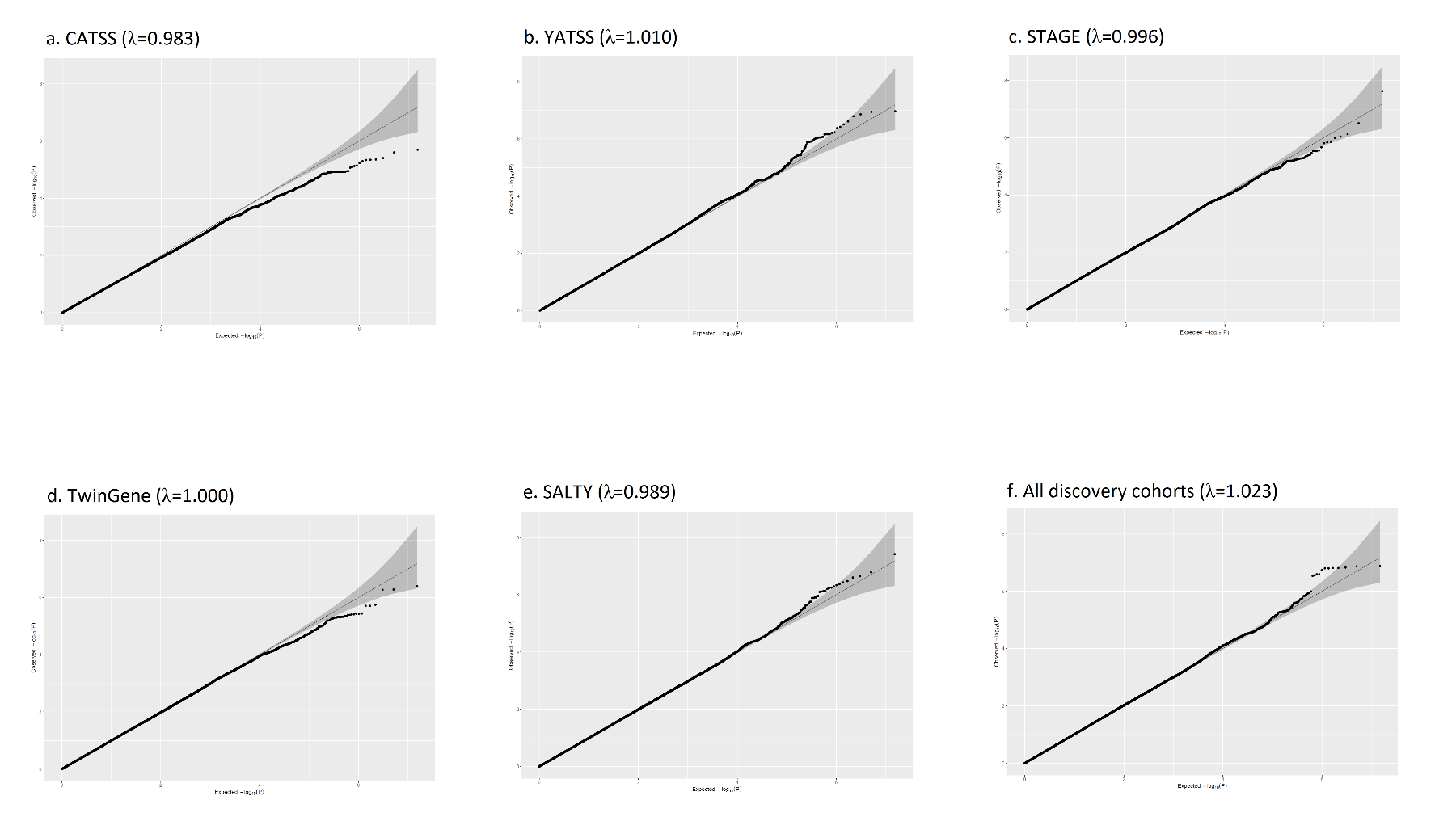


## Figure S3. Manhattan plots displaying GWAS results for dog ownership from the meta-analysis of discovery and replication cohorts separately. Upper panel represents meta-analyzed GWAS results of discovery cohorts and lower panel represents results from replication cohorts. Red and blue lines indicate the p-value thresholds at 5e-8 and 5e-7 respectively.


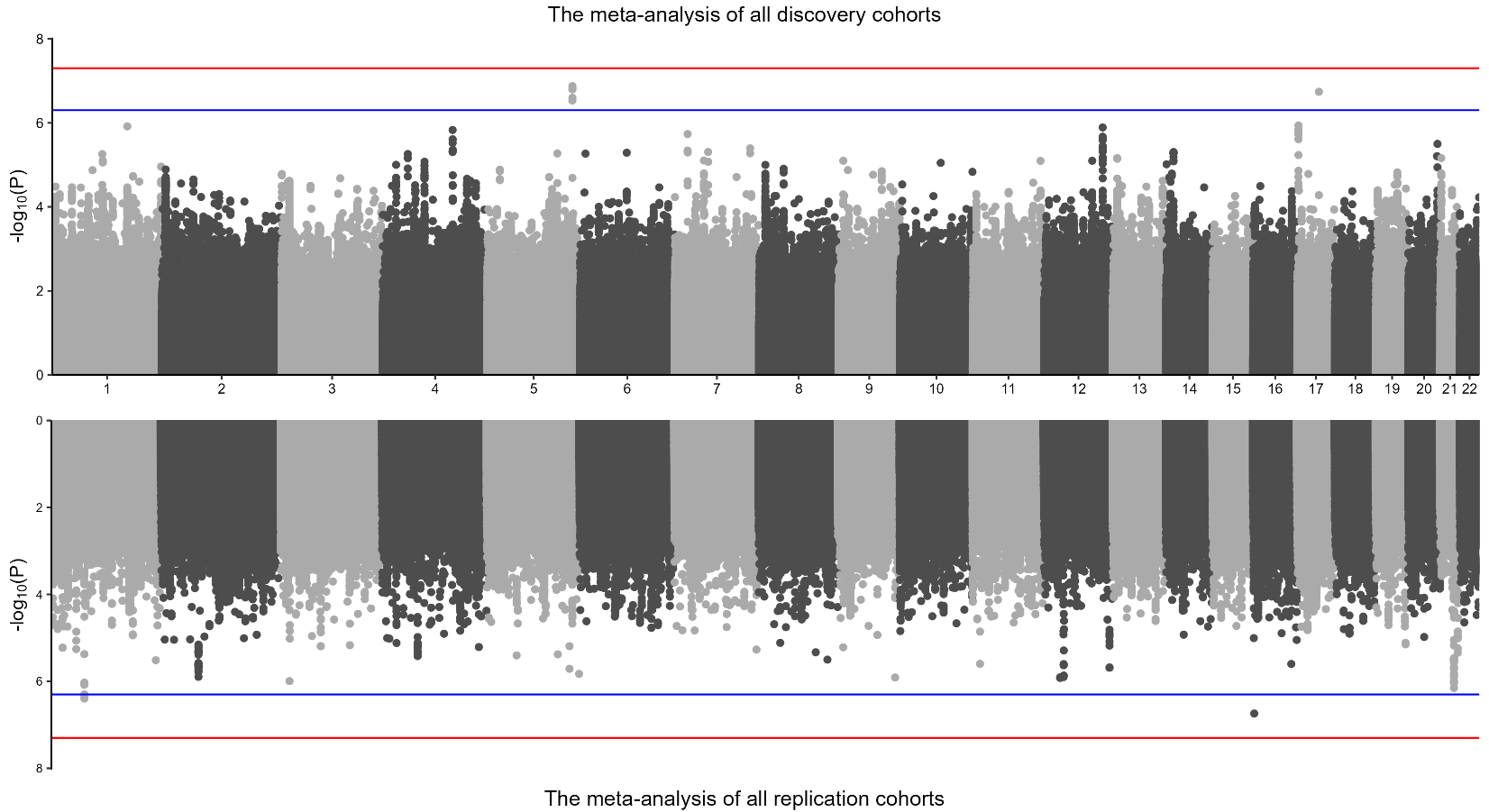


## Figure S4. Genetic correlation coefficients and 95% confidence intervals between dog ownership (using data from meta-analyzed discovery and replication cohorts) and 30 complex traits.


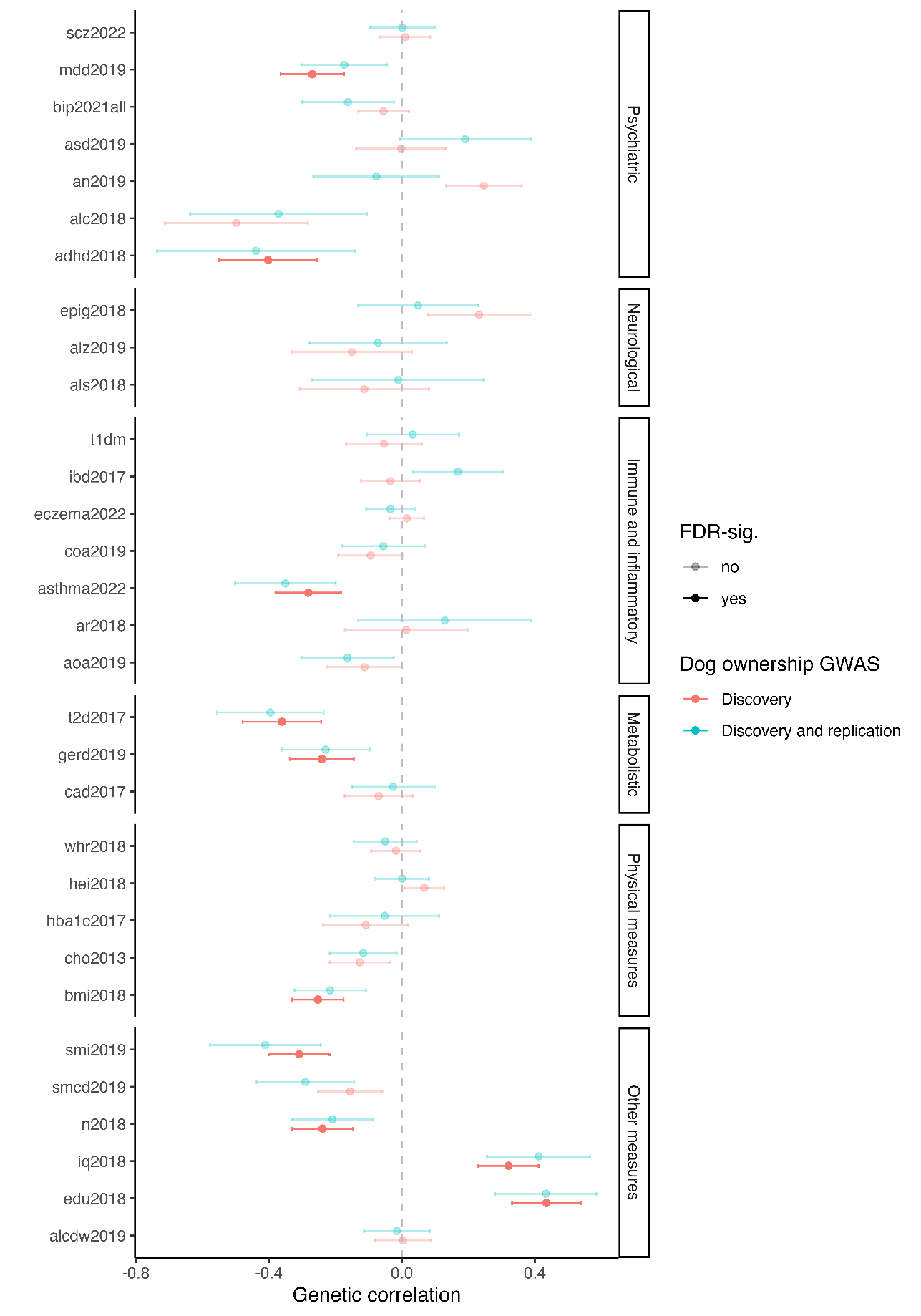


## Figure S5. Mahattan Plot of gene-based test


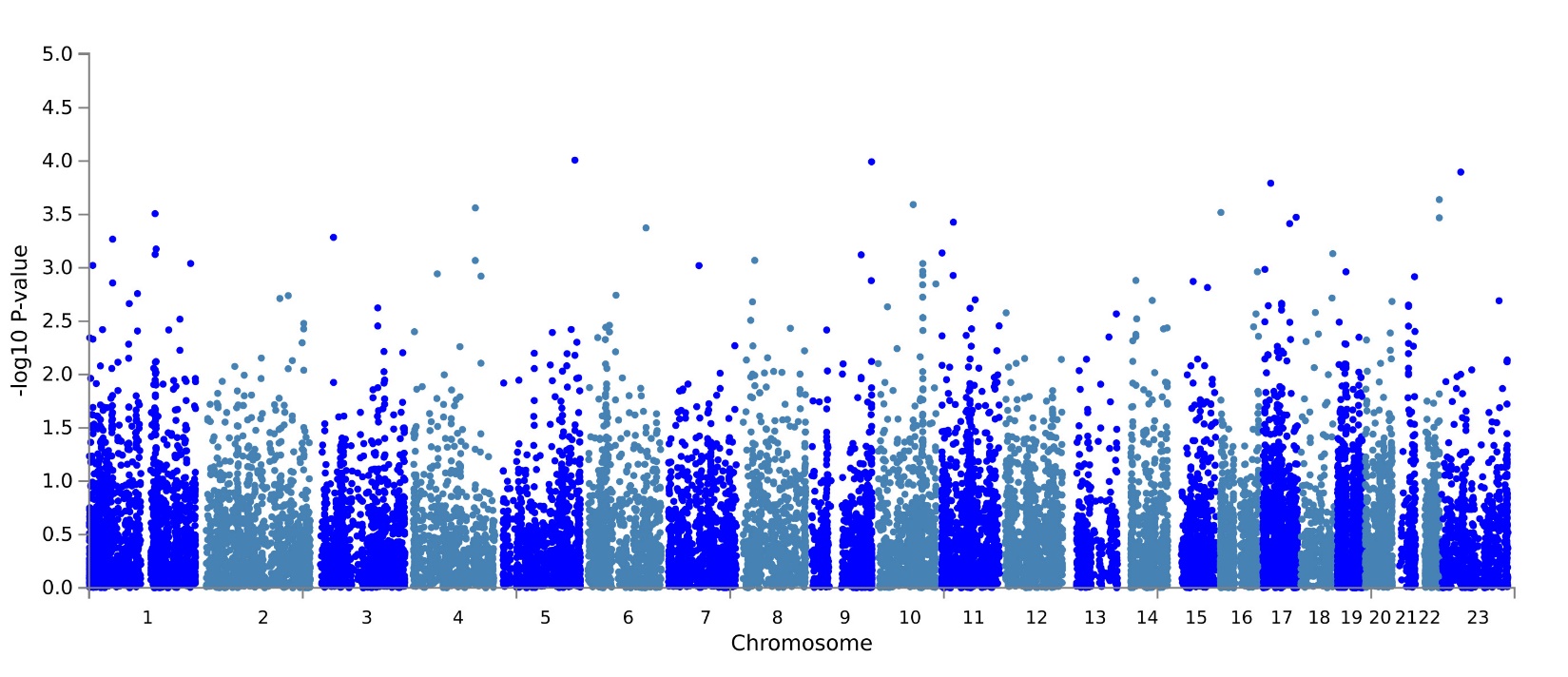


## Figure S6. Visualization of MAGMA tissue expression analysis.


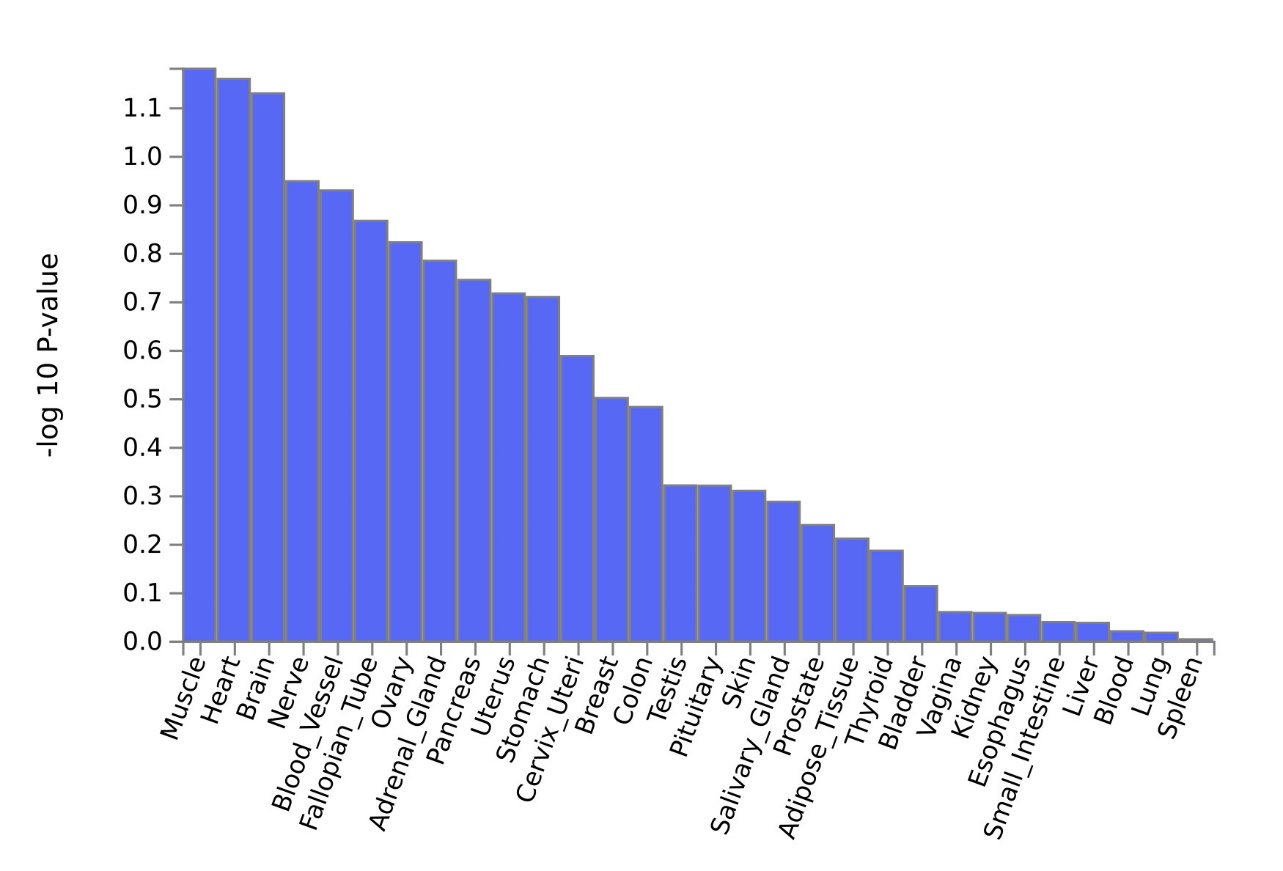


# **Readme for the summary statistics**

1. The summary statistics file contains columns present in the standard file format from METAL (see https://genome.sph.umich.edu/wiki/METAL_Documentation).

MarkerName – SNP name

Allele1 – Ref allele

Allele2 – Non-ref allele

Effect – Odds Ratio

StdErr – Standard error for odds ratio

P-value – p-value for the odds ratio

Direction – direction of the effect

HetISq – Heterogeneity statistics I^2^

HetChiSq - Heterogeneity statistics χ^2^

HetDf – Degrees of freedom in the statistical test for heterogeneity

HetPVAL – p-value for the statistical test for heterogeneity

TotalSampleSize – the sample size

2. The summary statistics file reports results of a genome-wide meta-analysis of dog onwership conducted in up to 97,552 European-ancestry individuals (from the Swedish Twin Registers, ELSA, BAMSE, HUNT).

# **Reference**

1. Purcell S, Neale B, Todd-Brown K, Thomas L, Ferreira MA, Bender D, et al. PLINK: a tool set for whole-genome association and population-based linkage analyses. Am J Hum Genet. 2007;81(3):559-75.

2. Danecek P, Auton A, Abecasis G, Albers CA, Banks E, DePristo MA, et al. The variant call format and VCFtools. Bioinformatics. 2011;27(15):2156-8.

3. Huff CD, Witherspoon DJ, Simonson TS, Xing J, Watkins WS, Zhang Y, et al. Maximum-likelihood estimation of recent shared ancestry (ERSA). Genome Res. 2011;21(5):768-74.

4. Laurie CC, Doheny KF, Mirel DB, Pugh EW, Bierut LJ, Bhangale T, et al. Quality control and quality assurance in genotypic data for genome-wide association studies. Genet Epidemiol. 2010;34(6):591-602.

5. Marees AT, de Kluiver H, Stringer S, Vorspan F, Curis E, Marie-Claire C, et al. A tutorial on conducting genome-wide association studies: Quality control and statistical analysis. Int J Methods Psychiatr Res. 2018;27(2):e1608.

6. Das S, Forer L, Schonherr S, Sidore C, Locke AE, Kwong A, et al. Next-generation genotype imputation service and methods. Nat Genet. 2016;48(10):1284-7.

7. Delaneau O, Zagury JF, Marchini J. Improved whole-chromosome phasing for disease and population genetic studies. Nat Methods. 2013;10(1):5-6.

8. Fuchsberger C, Abecasis GR, Hinds DA. minimac2: faster genotype imputation. Bioinformatics. 2015;31(5):782-4.

9. Howie B, Fuchsberger C, Stephens M, Marchini J, Abecasis GR. Fast and accurate genotype imputation in genome-wide association studies through pre-phasing. Nat Genet. 2012;44(8):955-9.

10. McCarthy S, Das S, Kretzschmar W, Delaneau O, Wood AR, Teumer A, et al. A reference panel of 64,976 haplotypes for genotype imputation. Nat Genet. 2016;48(10):1279-83.

11. Price AL, Patterson NJ, Plenge RM, Weinblatt ME, Shadick NA, Reich D. Principal components analysis corrects for stratification in genome-wide association studies. Nat Genet. 2006;38(8):904-9.

12. Genome-wide association study of 14,000 cases of seven common diseases and 3,000 shared controls. Nature. 2007;447(7145):661-78.

13. Åsvold BO, Langhammer A, Rehn TA, Kjelvik G, Grøntvedt TV, Sørgjerd EP, et al. Cohort Profile Update: The HUNT Study, Norway. medRxiv. 2021:2021.10.12.21264858.

14. Holmen J, Midthjell K, Krüger Ø, Langhammer A, Holmen TL, Bratberg GH, et al. The Nord-Trøndelag Health Study 1995-97 (HUNT 2): Objectives, contents, methods and participation. Norsk Epidemiologi. 2003;13(1):19-32.

15. Krokstad S, Langhammer A, Hveem K, Holmen TL, Midthjell K, Stene TR, et al. Cohort Profile: the HUNT Study, Norway. International journal of epidemiology. 2013;42(4):968-77.

16. Ferreira MA, Vonk JM, Baurecht H, Marenholz I, Tian C, Hoffman JD, et al. Shared genetic origin of asthma, hay fever and eczema elucidates allergic disease biology. Nat Genet. 2017;49(12):1752-7.

17. Brumpton BM, Graham S, Surakka I, Skogholt AH, Løset M, Fritsche LG, et al. The HUNT Study: a population-based cohort for genetic research. medRxiv. 2021:2021.12.23.21268305.

18. Wang C, Zhan X, Bragg-Gresham J, Kang HM, Stambolian D, Chew EY, et al. Ancestry estimation and control of population stratification for sequence-based association studies. Nat Genet. 2014;46(4):409-15.

19. Li JZ, Absher DM, Tang H, Southwick AM, Casto AM, Ramachandran S, et al. Worldwide human relationships inferred from genome-wide patterns of variation. Science. 2008;319(5866):1100-4.

20. Zhou W, Nielsen JB, Fritsche LG, Dey R, Gabrielsen ME, Wolford BN, et al. Efficiently controlling for case-control imbalance and sample relatedness in large-scale genetic association studies. Nat Genet. 2018;50(9):1335-41.
